# The Swedish version of the Bergen Social Media Addiction Scale: A psychometric evaluation among adolescents

**DOI:** 10.1016/j.abrep.2026.100704

**Published:** 2026-05-02

**Authors:** Malin Jakobsson, Gunilla Björling, Anders Broström, Karina Huus, Marit Eriksson, Staffan Bengtsson, Mark D. Griffiths, Amir Pakpour

**Affiliations:** a Department of Nursing, School of Health and Welfare, Jönköping University, Jönköping, Sweden; b CHILD-Research Group, Department of Nursing Science, School of Health and Welfare, Jönköping University, Jönköping, Sweden; c Department of Neurobiology Care Sciences and Society, Karolinska Institute, Stockholm, Sweden; d School of Nursing, KCMC University, Moshi, Tanzania; e Department of Clinical Neurophysiology, Linköping University Hospital, Linköping, Sweden; f Department of Health and Caring Sciences, Western Norway University of Applied Sciences, Bergen, Vestlandet, Norway; g Centre for Augmentative and Alternative Communication, University of Pretoria, Pretoria, South Africa; h Department of Health, Medicine and Caring Sciences, Linköping University, Linköping, Sweden; i Futurum- the Academy for Health and Care, Region Jönköping County, Jönköping, Sweden; j Department of Social Work, School of Health and Welfare, Jönköping University, Jönköping, Sweden; k Psychology Department, Nottingham Trent University, Nottingham, United Kingdom; l Division of Anesthesia, Operation, and Intensive Care, Södertälje Hospital, Södertälje, Sweden

**Keywords:** Problematic social media use, Social media addiction, Bergen Social Media Addiction Scale, Psychometric evaluation, Adolescents

## Abstract

**Background:**

The Bergen Social Media Addiction Scale (BSMAS) is one of the most widely used measures to assess problematic social media use (PSMU). However, no validated Swedish version for adolescents is available.

**Methods:**

The BSMAS was translated and culturally adapted into Swedish following established guidelines and psychometrically evaluated among a school-based sample of 658 adolescents aged 13–17 years from three schools (332 boys, 313 girls, and 13 with undisclosed gender; 580 Swedish-born and 78 non-Swedish-born). Reliability and construct validity were examined using classical test theory (CTT) and Rasch modeling. Confirmatory factor analysis (CFA) was conducted to assess dimensionality, and measurement invariance was tested across gender and country of birth. Evidence based on relationships to other variables was further explored using structural equation modeling and network analysis, including associations with social media use duration and symptoms of anxiety and depression assessed using the PROMIS Pediatric Depression and Anxiety Scales.

**Results:**

Among the competing CFA models, the best fit was obtained for a two-factor structure in which (i) salience and tolerance reflected peripheral criteria, and (ii) mood modification, relapse, withdrawal, and conflict reflected core criteria. Internal consistency was acceptable for both the peripheral factor (Cronbach’s α = 0.793; McDonald’s ω = 0.795) and the core factor (Cronbach’s α = 0.764; McDonald’s ω = 0.786). Rasch analyses indicated acceptable item fit and limited differential item functioning across gender and country of birth. Multigroup CFA supported configural, metric, and scalar invariance across both gender and country of birth. Network analyses showed that social media use duration was related to BSMAS symptoms but was not central in the symptom network. Structural equation modeling further showed that both peripheral and core criteria were positively associated with anxiety and depression, with stronger associations observed for core criteria.

**Conclusions:**

The Swedish version of the BSMAS showed acceptable reliability and validity among adolescents, with findings supporting a two-factor representation that distinguishes peripheral from core criteria. The scale appears suitable for adolescent research, school-based studies, and early screening contexts.

## Highlights

• The Swedish adolescent BSMAS supported a two-factor peripheral-core structure.
• The scale showed acceptable reliability and satisfactory item-level functioning.
• Measurement invariance was supported across gender and country of birth.
• Social media duration was linked to symptoms but was not central in the network.
• Core symptoms showed stronger links with anxiety and depressive symptoms.

## 1 Introduction

Recent estimates indicate that more than half of the world's population, over five billion people worldwide, use social media. The average daily time spent using social media is 140 min (Chaffey, 2025, Dixon, 2025). Adolescents are among the most active users, with the majority stating they use it almost constantly (Montag et al., 2024, Nagata et al., 2025). This highlights social media's ubiquity and relevance in adolescents' everyday lives. *Instagram, Facebook,* and *YouTube* are the most popular social media platforms across all ages (Chaffey, 2025, Dixon, 2025), together with *Snapchat* and *TikTok*, which are more common among younger users (Andersson et al., 2024, Nagata et al., 2025). These age-related differences in platform use may also be important for assessment, because emerging evidence suggests that associations between social media use and psychological difficulties may vary depending on patterns of use, contextual factors, and, potentially, the platforms involved (Kross et al., 2021, Valkenburg et al., 2022). Even adolescents under the minimum age required by most social media platforms (13 years) have social media accounts and use multiple platforms regularly (Nagata et al., 2025).

In Sweden, where the present study was conducted, social media use is highly prevalent among adolescents. Population-based survey data collected at the national level indicate that 97% of adolescents born in the 2000s and 82% of younger adolescents born in the 2010s use social media daily (Andersson et al., 2024). Longitudinal data from Swedish adolescents has indicated that median daily social media use was 1.7 h at baseline and increased over the subsequent three-year period (Beeres et al., 2021). Across the study waves, girls reported higher median daily use than boys (2.7 vs. 1.8 h/day). Importantly, these findings also suggested that time spent on social media alone had limited explanatory value for mental health outcomes, highlighting the need for validated instruments that assess problematic social media use (PSMU) rather than duration alone. Another Swedish study (Li Ma et al, 2021) found that spending more than two hours per day on social media was associated with higher odds of reporting depressive symptoms, further indicating the high prevalence and impact of social media use among Swedish adolescents.

These findings align with global patterns, where higher or problematic PSMU has shown negative associations with mental health, (e.g., anxiety, depression and stress) (Bozzola et al., 2022, Keles et al., 2019, Popat and Tarrant, 2022, Shannon et al., 2022), diet (related to junk food, obesity and unhealthy eating behaviors) (Bozzola et al., 2022), sleep impairment (Bozzola et al., 2022, Ahmed et al., 2024), academic achievement (Astatke et al., 2021, Salari et al., 2025), substance use and risky sexual behaviors (Vannucci et al., 2020), and emotional intelligence (e.g., self-esteem and emotional regulation) (Piccerillo and Digennaro, 2025).

Internalizing symptoms refer to inwardly directed emotional and somatic difficulties, including anxious, depressive, withdrawn, and somatic-complaint features (Achenbach et al., 2016). Among internalizing symptoms, depression and anxiety are among the most consistently examined correlates of PSMU. However, their temporal relationship with PSMU has not been definitively established as one of antecedence or consequence. In cross-sectional psychometric research, they are therefore best interpreted as theoretically relevant external correlates rather than as evidence of causal direction. Longitudinal and review evidence suggests that associations between social media use and depressive symptoms may be reciprocal in some studies, although the direction and mechanisms of these associations remain insufficiently established (Vidal et al., 2020). In a systematic review and meta-analysis focused on adolescents and young adults, PSMU was significantly associated with depressive symptoms, anxiety symptoms, and stress, with anxiety showing the strongest pooled association (Shannon et al., 2022). More recent meta-analytic work has likewise identified depression, anxiety, and stress as key psychological correlates of PSMU (Dadiotis et al., 2024). In addition, recent theory-driven work suggests that PSMU may, for some individuals, develop along a psychological-distress pathway in which avoidance expectancies and metacognitions about social compensation through social media use help explain the association between internalizing symptoms and problematic use (Casale et al., 2025). Together, these findings establish depression and anxiety as theoretically grounded external correlates against which PSMU measures can be evaluated and underscores the importance of identifying PSMU early in adolescence using psychometrically robust instruments.

Reliable and valid assessment tools are essential for accurately identifying and quantifying PSMU to inform research, prevention, and intervention efforts. Among the available instruments, the Bergen Social Media Addiction Scale (BSMAS) is one of the most widely used and psychometrically supported measures of PSMU. Although the scale title uses the term ‘addiction’, it is widely used to assess addiction-like symptoms within the broader construct of PSMU. Developed by Andreassen et al. (2016), the BSMAS is grounded in the components model of addiction (Griffiths, 2005), comprising salience, mood modification, tolerance, withdrawal, conflict, and relapse. The scale consists of six Likert-type items, making it brief, easy to administer, and suitable for large-scale surveys. Individuals rate the frequency of experiencing each symptom over the past year, with higher scores indicating greater risk of PSMU (Bányai et al., 2017).

Although most psychometric evaluations have found the BSMAS to be a unidimensional measure (Bottaro et al., 2025), some research suggests that not all six criteria may have equal clinical relevance in PSMU. Salience and tolerance have been proposed as more peripheral or engagement-related indicators, whereas relapse, withdrawal, and conflict appear to better reflect core problematic involvement. Recent adolescent evidence further suggests that a two-factor representation separating peripheral and core criteria may fit the BSMAS better than a single-factor solution (Ciudad-Fernández et al., 2025). In this study, the better-fitting model classified salience and tolerance as peripheral criteria, while mood modification, relapse, withdrawal, and conflict were classified as core criteria. Moreover, these two sets of criteria showed different associations with psychological well-being and distress. These findings raise the possibility that summing all six items into a single total score may, in some contexts, blur the distinction between high but non-problematic engagement and more clearly maladaptive use.

The BSMAS has been translated into multiple languages, including Italian (Monacis et al., 2017), Persian/Farsi (Lin et al., 2017, Sadeghi et al., 2025), Polish (Balcerowska et al., 2022), Canadian French (Rouleau et al., 2023), Greek (Dadiotis et al., 2021), Korean (Shin, 2022), Bengali (Naher et al., 2022), Chinese/Mandarin Chinese (Leung et al., 2020), Arabic (Abiddine et al., 2025, Al-Dassean and Murad, 2025), Romanian (Stănculescu, 2022), Slovenian (Žmavc et al., 2022), and Spanish (Arrivillaga et al., 2024), and validated in numerous cultural contexts. Earlier studies among Chinese-speaking populations also supported its psychometric robustness. For example, the Chinese BSMAS showed satisfactory psychometric properties and a unidimensional structure among Hong Kong university students (Yam et al., 2019). Similarly, among mainland Chinese primary school students, the simplified Chinese BSMAS demonstrated a unidimensional structure and invariance across gender (Chen et al., 2020a). In addition, longitudinal evidence from Chinese university students supported the time invariance of the BSMAS across a three-month interval, with satisfactory reproducibility (Chen et al., 2020b).

A recent meta-analysis, predominantly based on adult populations, summarized generally favorable psychometric evidence for the BSMAS, including strong internal consistency and broad support for a unidimensional structure (Bottaro et al., 2025). With evidence from 17 different languages, the meta-analysis confirmed BSMAS factor structure invariance and optimal internal consistency. Confirmatory factor analyses across diverse samples supported its construct validity, while correlations with measures of depression, anxiety, stress, and daily social media use provided evidence of concurrent validity (Bottaro et al., 2025; El Abiddine et al., 2025, Naher et al., 2022). Its brevity, ease of application, cross-cultural adaptability, and strong psychometric performance have established the BSMAS as a robust tool for assessing PSMU among both clinical and non-clinical populations (Yue et al., 2022).

Despite the extensive international use of the BSMAS, no adolescent-specific Swedish version had been specifically adapted and psychometrically evaluated prior to the present study. Although the BSMAS has previously been examined in a Swedish context, that evidence comes from a multinational adult study comprising participants aged 18 years and older and was not designed as an adolescent-focused Swedish adaptation (Brailovskaia & Margraf, 2024). This distinction is important because adolescents may interpret the wording of scale items differently from adults, and age-appropriate linguistic adaptation is needed before psychometric performance can be assumed to generalize across age groups.

Cultural and linguistic factors can influence how scale items are interpreted, potentially affecting the accuracy of responses and the validity of cross-cultural comparisons (Beaton et al., 2000, Guillemin et al., 1993). Direct translation without cultural adaptation may fail to capture nuances in meaning or relevance for specific populations, particularly adolescents, whose developmental stage influences their cognitive, emotional, and social engagement with social media (Arrivillaga et al., 2024, Montag et al., 2024). Given Sweden’s high prevalence of daily social media use among adolescents and documented associations between problematic use and adverse mental health outcomes (e.g., Keles et al., 2019), there is a clear need to adapt and validate the BSMAS for this group to ensure that it assesses PSMU accurately in the Swedish context.

Establishing the psychometric properties of a Swedish BSMAS, such as reliability, factor structure, measurement invariance, and concurrent validity, will allow researchers and clinicians to confidently use the scale for screening, monitoring, and cross-national research. Moreover, a validated Swedish version among adolescents will facilitate early detection and preventive efforts related to PSMU, thereby addressing both clinical needs and broader public health concerns (Andreassen, 2015, Bányai et al., 2017). Although previous work has examined the BSMAS in Sweden (Brailovskaia & Margraf, 2024), existing Swedish evidence is based on adult sample (18 + years) recruited online (e.g., online panels/volunteer web surveys), which may not generalize to school-based adolescent populations.

Therefore, the present study linguistically reviewed, culturally adapted, and psychometrically evaluated an adolescent-specific Swedish version of the BSMAS. More specifically, the study examined its internal consistency, item functioning, latent structure, measurement invariance across gender and country of birth, and associations with relevant external variables. It was hypothesized that the Swedish BSMAS would (i) demonstrate acceptable reliability and satisfactory item functioning (H_1_), and (ii) show measurement invariance across gender and country of birth (H_2_), and (iii) provide evidence based on relationships to other variables (convergent/concurrent validity), specifically positive associations with social media use duration, anxiety, and depressive symptoms (H_3_).

Because earlier psychometric studies often supported a unidimensional structure of the BSMAS (e.g., Bányai et al., 2017, Lin et al., 2017), whereas more recent work has challenged this assumption by suggesting a distinction between peripheral and core criteria (Fournier et al., 2023, Fournier et al., 2024, Ciudad-Fernández et al., 2025), the latent structure was treated as a theory-informed research question and evaluated by comparing competing CFA models. In addition, given emerging evidence that the BSMAS criteria may not function as fully interchangeable indicators of PSMU, symptom-level network analyses were conducted to examine whether individual items showed differential associations with social media use duration and internalizing symptoms.

## 2 Methods

### 2.1 Design

To address the aim of the present study, a multi-step methodological approach was employed. First, the Swedish wording of the BSMAS was linguistically reviewed and culturally adapted for adolescent use in accordance with established guidelines (Beaton et al., 2000). Thereafter, a cross-sectional survey design was used to evaluate the psychometric properties of the Swedish BSMAS among adolescents.

### 2.2 Translation procedure

The BSMAS was translated and culturally adapted into Swedish using a structured multistep procedure. This included independent forward translations, synthesis of the translations, blind back translation, expert committee review, and pilot cognitive testing with adolescents to ensure semantic equivalence, cultural relevance, and age-appropriate comprehension. Minor wording refinements were made following pilot testing, and the finalized version was used in the present study. Detailed information on the translation and adaptation procedure is provided in the Supplementary Materials (Appendix 1).

### 2.3 Participants and procedure

Data were collected from adolescents recruited from three secondary schools in western and eastern Sweden. The survey was administered online during mentor periods in the classrooms. Of the 1,048 students across the three schools, 658 adolescents had parental consent (required for those under the age of 15 years) and had given their own consent and were thereby included in the analysis. The sample comprised participants aged 13 to 17 years (332 boys, 313 girls, and 13 who chose not to disclose their gender; 580 Swedish-born and 78 non-Swedish-born). All participants reported using social media.

### 2.4 Measures

#### 2.4.1 Bergen Social Media Addiction Scale (BSMAS)

As aforementioned, the BSMAS (Andreassen et al., 2016) is a brief six-item self-report instrument designed to assess symptoms of PSMU over the past year. Each item (e.g., *“You feel an urge to use social media more and more”*) corresponds to one of six core addiction components: salience, mood modification, tolerance, withdrawal, conflict, and relapse. Participants respond on a five-point Likert scale ranging from 1 (*very rarely*) to 5 (*very often*), with higher total scores (range 6–30) indicating greater risk of PSMU.

#### 2.4.2 PROMIS Pediatric Anxiety Short Form

Anxiety symptoms were assessed using the PROMIS Pediatric Anxiety Short Form v2.0 (Irwin et al., 2010). This scale consists of eight items assessing symptoms of anxiety experienced in the past seven days, including feelings of nervousness, worry, and fear. Items (e.g., *“I felt like something awful might happen”*) are rated on a five-point Likert scale ranging from 1 (*never*) to 5 (*almost always*). Total raw scores range from 8 to 40, with higher total scores indicating more severe anxiety symptoms (Blomqvist et al., 2025). The raw scores were used for analysis. In the present study, the internal consistency was excellent (Cronbach’s α = 0.936).

#### 2.4.3 PROMIS Pediatric Depressive Symptoms Short Form

Depressive symptoms were assessed using a modified seven-item version of the PROMIS Pediatric Depressive Symptoms Short Form v2.0 (Blomqvist et al., 2025). One item from the original eight-item form (*“I felt lonely”*) was omitted in the present study because it was judged by the research team to be highly overlapping in meaning with another retained item (*“I felt alone”*). To reduce content redundancy and minimize burden on participants in the school-based context, only one of these two semantically near-identical items was retained. The seven items assess feelings of sadness, loneliness, lack of enjoyment, and hopelessness experienced during the past seven days. Items are rated on a five-point Likert scale from 1 (*never*) to 5 (*almost always*) (Blomqvist et al., 2025). Raw scores range from 7 to 35, with higher total scores indicating greater depressive symptom severity. Due to the omission of one item, raw scores were used instead of T-scores. In the present study, the internal consistency was excellent (α = 0.945).

### 2.5 Data analysis

All statistical analyses were conducted using *IBM SPSS Statistics version 27, Winsteps version 4.3.0*, and *JASP software version 0.19.3.0*. The significance level was set at *p* < 0.05 for all tests. The psychometric evaluation of the Swedish BSMAS was organized according to contemporary validity theory, particularly the sources of validity evidence outlined in the *Standards for Educational and Psychological Testing* (American Educational Research Association, American Psychological Association, & National Council on Measurement in Education, 2014; see also Furr, 2021). Evidence based on internal structure was examined through confirmatory factor analyses and tests of measurement invariance. Reliability and item-level functioning were evaluated using Cronbach’s α, McDonald’s ω, item–total correlations, and Rasch modeling.

Evidence based on relationships to other variables was examined through structural equation modeling and network analyses assessing associations of BSMAS dimensions and items with anxiety symptoms, depressive symptoms, and self-reported daily time spent on social media. Anxiety and depressive symptoms were treated as theoretically expected internalizing correlates of PSMU and therefore as evidence relevant to convergent validity. Self-reported time spent on social media was treated as a concurrent external behavioral correlate. However, because self-reported duration is an imperfect indicator of actual social media use and tends to diverge from log-based measures (Parry et al., 2021), it was interpreted as a coarse behavioral correlate rather than as an objective exposure measure.

#### 2.5.1 Reliability

Internal consistency of the BSMAS was evaluated using both Cronbach’s alpha and McDonald’s omega coefficients. Values above 0.70 were considered acceptable. The internal consistency was further examined by computing corrected item-total correlation coefficients to evaluate how well each item correlated with the overall scale score excluding that item. Items with a correlation of 0.40 or higher were considered as making an acceptable contribution to the BSMAS’s internal consistency (Cicchetti, 1994).

#### 2.5.2 Construct validity

The factor structure of the Swedish BSMAS was evaluated using confirmatory factor analysis (CFA) with the Weighted Least Squares Means and Variance adjusted (WLSMV) estimator, which is appropriate for ordinal Likert-scale data. In addition to the original one-factor model, supplementary CFA models were estimated to address recent debate regarding whether the BSMAS may distinguish more peripheral from more core criteria (Ciudad-Fernández et al., 2025). More specifically, three models were compared: (i) a unidimensional model including all six items loading on a single latent factor; (ii) a two-factor model in which salience, tolerance, and mood modification were specified as peripheral criteria, and relapse, withdrawal, and conflict were specified as core criteria; and (iii) a two-factor model in which salience and tolerance were specified as peripheral criteria, and mood modification, relapse, withdrawal, and conflict were specified as core criteria.

The following model fit indices were evaluated to assess the adequacy of the factor model: chi-square test (χ^2^) and degrees of freedom (df), Comparative Fit Index (CFI), Tucker-Lewis Index (TLI), Root Mean Square Error of Approximation (RMSEA), and Standardized Root Mean Square Residual (SRMR). An acceptable model typically shows a nonsignificant χ^2^, CFI and TLI values greater than 0.95, RMSEA less than 0.06, and SRMR less than 0.08 (Bowen and Masa, 2015, Hooper et al., 2008). Model comparisons were interpreted descriptively based on overall fit indices.

#### 2.5.3 Measurement invariance

Multi-group CFA was performed to evaluate measurement invariance of the BSMAS across gender and country of birth (Swedish born vs. non-Swedish born). Configural (same factor structure), metric (equal factor loadings), and scalar (equal factor loadings and thresholds) invariance were tested sequentially. Differences in CFI (ΔCFI ≤ 0.01), RMSEA (ΔRMSEA ≤ 0.03) and SRMR (ΔSRMR < 0.01) between nested models were used as criteria for invariance (Patrick, 2019, Sick, 2008).

#### 2.5.4 Rasch analysis

To further evaluate the psychometric properties of the Swedish BSMAS at the item level, Rasch analysis was conducted using *Winsteps version 4.3.0*. The Rating Scale Model (RSM) was applied given that all items shared the same Likert-type response format, allowing for the assessment of item difficulty and threshold parameters on a common rating scale structure. Key fit statistics, including infit and outfit mean square (MnSq) values, were examined to assess how well each item fitted the Rasch model. Values between 0.7 and 1.3 are considered acceptable, indicating good item fit; values outside this range suggest potential misfit (Tesio, 2003).

To evaluate the invariance of the BSMAS items across gender and country of birth groups, Differential Item Functioning (DIF) was computed. A DIF contrast (difference in item difficulty between groups) of less than 0.5 logits is considered negligible, indicating measurement invariance. Moreover, person and item separation indices and corresponding reliability coefficients were reported to indicate the scale’s precision and discriminative ability. Separation indices greater than 2.0 and reliability values above 0.80 are considered indicators of good measurement precision and ability to distinguish between different levels of the latent trait. To evaluate the assumption of unidimensionality, a principal component analysis (PCA) of residuals was performed. The variance explained by the Rasch dimension should be greater than 50%, and the eigenvalue of the first contrast in the residuals should be less than 2.0, supporting unidimensionality (Linacre, 2012). Potential local dependency among items was investigated by examining residual correlations. Residual correlations above 0.20 indicate local dependence, suggesting that items share variance beyond the latent trait, which may violate Rasch model assumptions.

#### 2.5.5 Structural equation modeling

To further examine evidence based on relationships to other variables, structural equation modeling (SEM) was conducted in *R* using the *lavaan* package. The analyses were based on the two-factor BSMAS model in which BSMAS1 and BSMAS2 represented peripheral criteria and BSMAS3 to BSMAS6 represented core criteria. Anxiety and depression were modelled as latent endogenous factors using the PROMIS anxiety and depressive symptom items. Because all items were ordinal, models were estimated using the WLSMV estimator. Model fit was evaluated using χ^2^, CFI, TLI, RMSEA, and SRMR. First, a latent correlation model was estimated to examine associations among peripheral criteria, core criteria, anxiety, and depression. Thereafter, SEMs were estimated to examine the associations of peripheral and core criteria with anxiety and depression. Because the peripheral and core factors were highly correlated in the joint model, separate SEMs were also estimated for each BSMAS factor to obtain more interpretable parameter estimates.

#### 2.5.6 Network analysis

Two separate network analyses were conducted to examine evidence based on relationships between BSMAS items and theoretically relevant external variables. Average daily social media duration was included as a behavioral correlate, whereas depressive and anxiety symptoms were included as internalizing correlates based on prior evidence associating PSMU with psychological distress and internalizing symptoms (Casale et al., 2025, Dadiotis et al., 2024, Shannon et al., 2022;).

Behavioral correlate network: To examine associations between individual BSMAS items and average daily social media duration (measured in hours), a network was estimated including the six BSMAS items and daily duration of social media use. This analysis was intended to evaluate how the symptom indicators relate to actual reported use behavior.

Internalizing correlates network: To examine associations between BSMAS symptoms and internalizing distress, a network was estimated including all BSMAS items together with items from the PROMIS Pediatric Anxiety and Depressive Symptoms short forms.

Network estimation and visualization: Networks were estimated using the Graphical Least Absolute Shrinkage and Selection Operator (glasso) method combined with the Extended Bayesian Information Criterion (EBIC) model selection procedure. This approach produces sparse and interpretable networks by penalizing weaker edges, effectively controlling spurious associations through shrinking small partial correlations toward zero.

In the resulting networks, nodes represent individual scale items, and edges represent the strength of partial correlations between these items, controlling all other variables in the network. To understand the importance of individual symptoms, measures of centrality such as strength, closeness, and betweenness were calculated, highlighting the most influential symptoms within each network. The stability and accuracy of the estimated networks and centrality measures were assessed through 500 bootstrap iterations, ensuring the robustness and reliability of the findings.

### 2.6 Ethics

Ethical research principles were carefully followed by fulfilling the requirements of respect for autonomy, nonmaleficence, beneficence, and justice (Swedish Research Council, 2024). To ensure ethical principles were followed, the participants received oral and written information about the aim of the study before the survey. The students were also informed about their rights and that participation was voluntary. Consent was obtained from all participants. Adolescents under the age of 15 years also had consent from their parents. These procedures ensured the provision of clear and sufficient information, the obtaining of informed consent, the protection of confidentiality, and the appropriate use of collected data, in full compliance with the ethical standards outlined in the Declaration of Helsinki (World Medical Association, 2013). Ethical approval for the project was granted by the Swedish Ethical Review Authority (Dnr 2024–02213-01 and Dnr 2024–04153-02).

## 3 Results

### 3.1 Construct validity

Three competing CFA models were estimated to evaluate the dimensionality of the Swedish BSMAS. The unidimensional model showed suboptimal fit, χ^2^(9) = 65.30, *p* < 0.001, CFI = 0.982, TLI = 0.970, RMSEA = 0.100, and SRMR = 0.041. The first two-factor model, in which salience, tolerance, and mood modification were specified as peripheral criteria and relapse, withdrawal, and conflict as core criteria, showed somewhat improved but still suboptimal fit, χ^2^(8) = 50.80, *p* < 0.001, CFI = 0.986, TLI = 0.974, RMSEA = 0.092, and SRMR = 0.035. In contrast, the second two-factor model, in which salience and tolerance were specified as peripheral criteria and mood modification, relapse, withdrawal, and conflict as core criteria, showed the best fit, χ^2^(8) = 10.85, p = 0.211, CFI = 0.999, TLI = 0.998, RMSEA = 0.024, and SRMR = 0.018. Accordingly, this two-factor model was retained as the preferred representation of the BSMAS in the present sample. The correlation between the peripheral and core factors in this model was 0.859 (*p* < 0.001), indicating that the two latent dimensions were strongly related but empirically distinguishable. Standardized factor loadings in the preferred model ranged from 0.721 to 0.897 (Table 1, Table 2).

**Table 1:** Confirmatory factor analysis models.

Models	χ^2^	df	*p*-value	CFI	TLI	RMSEA	SRMR
Model 1: unidimensional	65.30	9	< 0.001	0.982	0.970	0.100	0.041
Model 2: Two-factor (1,2,3 peripheral; 4,5,6 core)	50.80	8	< 0.001	0.986	0.974	0.092	0.035
Model 3: Two-factor (1,2 peripheral; 3,4,5,6 core)	10.85	8	0.211	0.999	0.998	0.024	0.018

**Table 2:** Psychometric properties of the Bergen Social Media Addiction Scale (BSMAS) at item level.

**Item number**	**Analyses from Classical Test Theory**		**Rasch Analyses**				
	Factor loading *^†^	**Item–total correlation**	**Infit MnSq**	**Outfit MnSq**	Difficulty	DIF contrast across gender ^a¶^	DIF contrast across place of birth ^b¶^
BSMAS1	0.830	0.661	0.95	0.95	−0.55	−0.07	−0.07
BSMAS2	0.897	0.661	1.02	1.01	0.55	0.07	0.07
BSMAS3	0.783	0.589	0.99	0.96	−0.93	−0.51	0.15
BSMAS4	0.721	0.580	1.07	1.07	−0.25	0.20	−0.10
BSMAS5	0.855	0.599	0.91	0.84	1.15	−0.04	0.34
BSMAS6	0.759	0.609	0.97	1.03	0.04	0.45	−0.33

^a^Gender: girls – boys.

^b^Country of birth: Swedish born − non-Swedish born.

### 3.2 Measurement invariance

Multi-group CFA was used to examine measurement invariance of the preferred two-factor BSMAS model across gender and country of birth. Across gender, the configural model showed good fit: χ^2^(16) = 21.885, CFI = 0.998, SRMR = 0.025, and RMSEA = 0.035. Constraining factor loadings to equality across groups did not significantly worsen model fit: robust Δχ^2^(4) = 5.465, *p* = 0.243, with negligible changes in approximate fit indices (ΔCFI =  − 0.001, ΔSRMR = +.008, ΔRMSEA =  − 0.001). Moreover, further constraining thresholds did not significantly worsen fit: robust Δχ^2^(12) = 10.017, *p* = 0.615, with no meaningful decline in fit (ΔCFI = 0.000, ΔSRMR =  − 0.006, ΔRMSEA =  − 0.008). These findings supported configural, metric, and scalar invariance across gender.

Similarly, across country of birth, the configural model showed good fit: χ^2^(16) = 21.227, CFI = 0.998, SRMR = 0.024, and RMSEA = 0.033. Equality constraints on factor loadings did not significantly worsen fit: robust Δχ^2^(4) = 1.917, *p* = 0.751. Moreover, adding threshold constraints did not significantly worsen fit: robust Δχ^2^(11) = 15.048, *p* = 0.180. Changes in CFI, SRMR, and RMSEA were again trivial. Therefore, the preferred two-factor structure demonstrated configural, metric, and scalar invariance across country of birth. For the country-of-birth analysis, the two highest response categories of BSMAS5 were collapsed because one top category was empty in one group (Table 3).

**Table 3:** Measurement invariance of the two-factor structure of the Bergen Social Media Addiction Scale (BSMAS) through confirmatory factor analysis.

Model and comparisons	χ^2^ (df)	Δχ^2^ (Δdf)	CFI	ΔCFI	SRMR	ΔSRMR	RMSEA	ΔRMSEA	Robust difference test *p*-value
Gender									
M1: Configural	21.885 (16)	—	0.998	—	0.025	—	0.035	—	—
M2: Metric	26.945 (20)	—	0.997	−0.001	0.033	0+.008	0.034	−0.001	—
M3: Scalar	38.700 (32)	—	0.997	0.000	0.027	−0.006	0.026	−0.008	—
M2 − M1	—	5.465 (4)	—	−0.001	—	0+.008	—	−0.001	0.243
M3 − M2	—	10.017 (12)	—	0.000	—	−0.006	—	−0.008	0.615
Country of birth									
M1: Configural	21.227 (16)	—	0.998	—	0.024	—	0.033	—	—
M2: Metric	20.838 (20)	—	1.000	0+.002	0.025	0+.001	0.012	−0.021	—
M3: Scalar	38.473 (31)	—	0.998	−0.002	0.024	−0.001	0.028	0+.016	—
M2 − M1	—	1.917 (4)	—	0+.002	—	0+.001	—	−0.021	0.751
M3 − M2	—	15.048 (11)	—	−0.002	—	−0.001	—	0+.016	0.180

CFI = comparative fit index; SRMR = standardized root mean square residual; RMSEA = root mean square error of approximation

Note. M1 = configural model; M2 = metric model; M3 = scalar model. For ordered categorical items, scalar invariance refers to equality of factor loadings and thresholds across groups. The per-model χ^2^ values are the scaled statistics from the lavaan model summaries, whereas the Δχ^2^ values are the robust scaled chi-square difference tests from lavTestLRT(). Therefore, the reported Δχ^2^ values are not expected to equal the simple arithmetic difference between the model χ^2^ values.

### 3.3 Reliability

The preferred two-factor structure of the Swedish BSMAS demonstrated acceptable internal consistency at the scale level. For the peripheral factor (BSMAS1 and BSMAS2), Cronbach’s α was 0.793, McDonald’s ω was 0.795, and the average variance extracted (AVE) was 0.747. For the core factor (BSMAS3 to BSMAS6), Cronbach’s α was 0.764, McDonald’s ω was 0.786, and the AVE was 0.610. At the item level, item–total correlations ranged from 0.580 to 0.661, indicating that all six items were meaningfully related to the overall construct (Table 4).

**Table 4:** Psychometric properties of the Bergen Social Media Addiction Scale (BSMAS) at scale level.

Psychometric testing	BSMAS peripheral (Factor 1)	BSMAS core (Factor 2)
Cronbach’s α Internal consistency	0.793	0.764
McDonald’s omega	0.795	0.786
Average Variance Extracted	0.747	0.610
Item separation reliability from Rasch	0.97	0.99
Item separation index from Rasch	5.61	10.63
Person separation reliability from Rasch	0.68	0.44
Person separation index from Rasch	1.47	0.88

### 3.4 Rasch analysis

Rasch modelling using the Rating Scale Model showed acceptable item fit for all six BSMAS items. Infit MnSq values ranged from 0.91 to 1.07, and outfit MnSq values ranged from 0.84 to 1.07, indicating that all items fitted the Rasch model satisfactorily. Item difficulty estimates ranged from − 0.93 logits for BSMAS3 to 1.15 logits for BSMAS5, suggesting that the items covered a meaningful range of symptom severity. DIF contrasts across country of birth were small for all items, ranging from − 0.33 to 0.34. DIF contrasts across gender were also generally small, ranging from − 0.51 to 0.45, although BSMAS3 showed a marginally elevated contrast (−0.51).

At the scale level, Rasch item separation reliability was high for both factors (0.97 for the peripheral factor and 0.99 for the core factor), with corresponding item separation indices of 5.61 and 10.63, indicating good differentiation of item difficulty. In contrast, person separation reliability was lower (0.68 for the peripheral factor and 0.44 for the core factor), with person separation indices of 1.47 and 0.88, suggesting that the scale differentiated item difficulty more clearly than respondent trait levels, especially for the core dimension (Table 2, Table 4).

### 3.5 Structural equation modeling

The SEM based on the two-factor BSMAS structure showed acceptable fit according to scaled indices, χ^2^(183) = 587.72, CFI = 0.986, TLI = 0.984, RMSEA = 0.062 (90% CI [.056, 0.067]), and SRMR = 0.039. The latent correlation model showed that both BSMAS dimensions were positively associated with internalizing symptoms. Peripheral criteria were moderately correlated with anxiety (*r* = 0.46) and depression (*r* = 0.48), whereas core criteria showed stronger correlations with anxiety (*r* = 0.69) and depression (*r* = 0.71). The correlation between peripheral and core criteria was high (*r* = 0.85).

Because the strong overlap between peripheral and core criteria produced unstable coefficients in the joint SEM, separate SEMs were also estimated. The peripheral-only model showed acceptable scaled fit: χ^2^(116) = 434.51, CFI = 0.988, TLI = 0.986, RMSEA = 0.068 (90% CI [.062, 0.075]), SRMR = 0.034. In this model, peripheral criteria were positively associated with anxiety (β = 0.47, *p* < 0.001) and depression (β = 0.48, *p* < 0.001). The core-only model also showed acceptable scaled fit: χ^2^(149) = 543.55, CFI = 0.985, TLI = 0.983, RMSEA = 0.067 (90% CI [.061, 0.074]), SRMR = 0.038. In this model, core criteria were positively associated with anxiety (β = 0.67, *p* < 0.001) and depression (β = 0.69, *p* < 0.001). Overall, core criteria showed stronger associations with both anxiety and depression than peripheral criteria.

### 3.6 Network analysis

#### 3.6.1 Behavioral correlate network

**Fig. 1:**
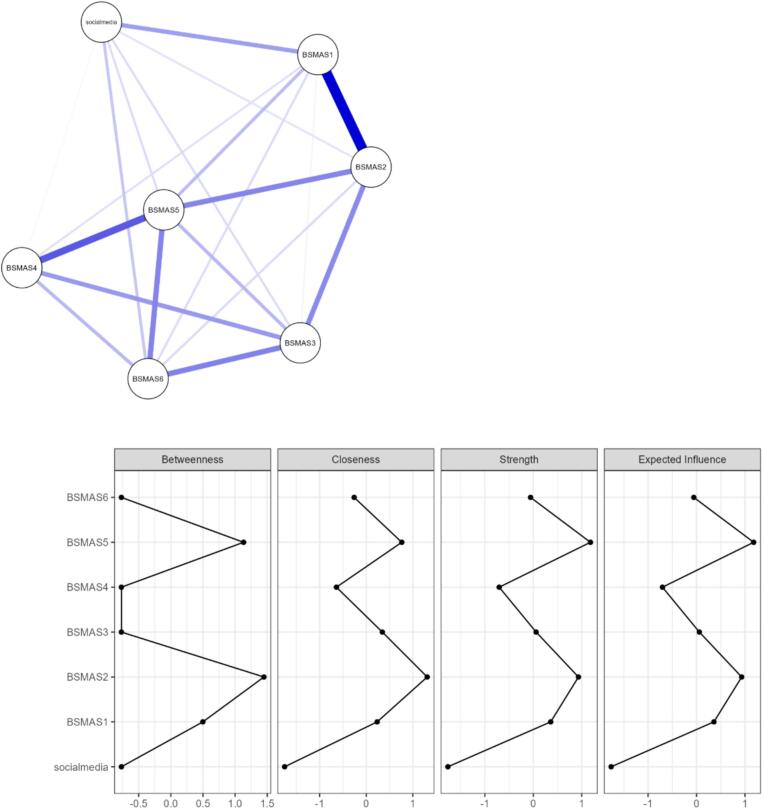
Network structure and centrality indices of the BSMAS items and social media duration. (A) Network of partial correlations among BSMAS items and social media duration. Nodes represent individual items; edges represent partial correlations adjusted for all other variables. Thicker and darker edges indicate stronger associations. (B) Centrality indices (betweenness, closeness, strength, and expected influence) for each node, reflecting their relative importance in the network.

Network analysis assessed the direct partial correlations between individual BSMAS items and average daily social media duration (hours). The network comprised seven nodes (six BSMAS items and social media duration) and 20 non-zero edges, with low sparsity (0.048). The strongest edge connecting a BSMAS item to social media duration was observed for BSMAS Item 1 (*“Spent a lot of time thinking about social media or planning how to use it”*) (edge weight = 0.182). Other notable connections included BSMAS Item 6 *([“You use social media so much that it has had a negative impact on your job/studies”*] edge weight = 0.108) and Item 5 ([*“You become restless or troubled if you are prohibited from using social media”*] edge weight = 0.068) with duration of social media use. Notably, social media duration itself showed modest overall centrality, suggesting it is influenced by these symptoms but not central to the network (Fig. 1).

#### 3.6.2 Internalizing correlates network

**Fig. 2:**
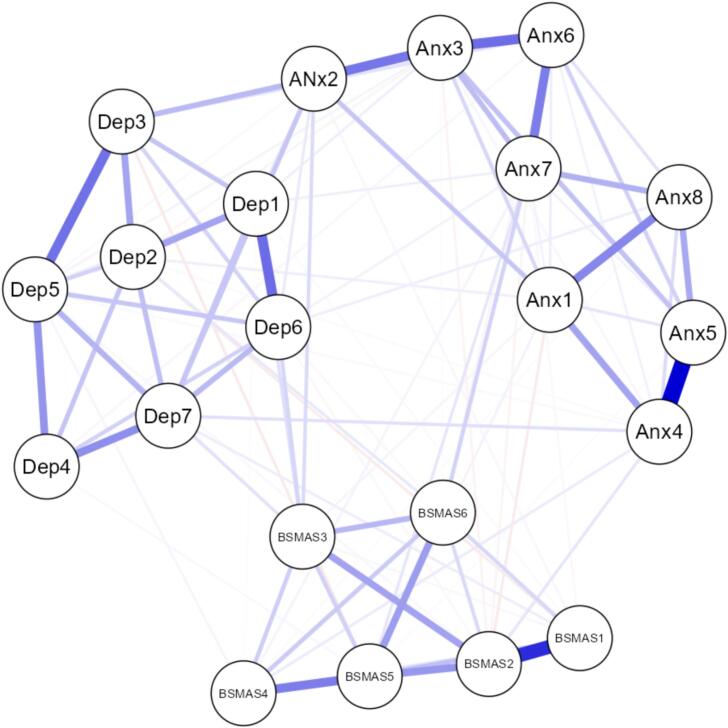

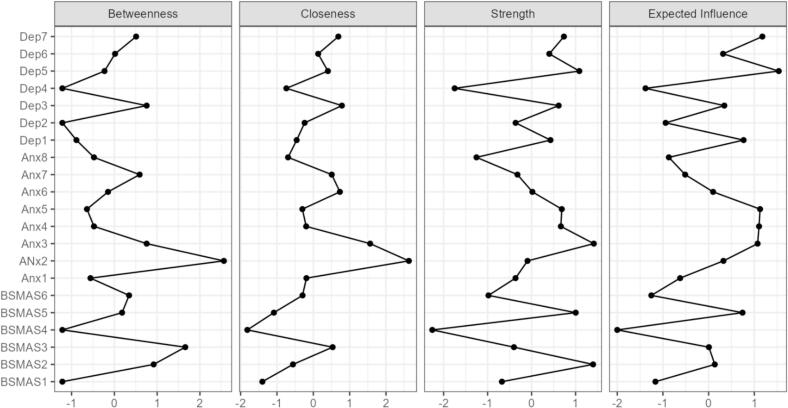
Network structure and centrality indices of BSMAS, anxiety, and depression items. (A) Network of partial correlations among BSMAS items, PROMIS Pediatric Anxiety items, and PROMIS Pediatric Depression items. Nodes represent individual symptoms; edges represent partial correlations adjusted for all other variables. Thicker and darker edges indicate stronger associations. (B) Centrality indices (betweenness, closeness, strength, and expected influence) for each node, indicating their relative importance and connectivity within the network.

A second network analysis examined the partial correlations among all BSMAS items and items from the PROMIS Pediatric Anxiety (eight items) and Depression (seven items) short forms. The network consisted of 21 nodes and 107 non-zero edges, with a sparsity of 0.49. The strongest edge connecting a BSMAS item to an anxiety or depression symptom was observed between BSMAS Item 6 (*“use social media so much that it has had a negative impact on studies”*) and anxiety Item 1 (*“felt that something terrible might happen”*), with an edge weight of approximately 0.293. Other notable edges included BSMAS Item 2 (*“feel an urge to use social media more and more”*) with anxiety Item 2 (*“felt anxiety”*, edge weight = 0.274) and BSMAS Item 1 (*“spent a lot of time thinking about social media or planning how to use it”*) with depression Item 3 (*“felt sad”,* edge weight = 0.158) (Fig. 2).

## 4 Discussion

The present study translated, culturally adapted, and psychometrically evaluated an adolescent-specific Swedish version of the BSMAS. Overall, the findings indicated that the Swedish BSMAS demonstrated acceptable internal consistency (supporting H_1_), satisfactory item-level performance, and measurement invariance across gender and country of birth (supporting H_2_). The findings also supported H_3_. BSMAS symptoms were positively associated with social media use duration, anxiety, and depressive symptoms, although these associations were stronger and more clinically meaningful for the core criteria than for the peripheral criteria. Regarding dimensionality, the results did not support retaining a strictly unidimensional interpretation in this particular sample. Instead, the best-fitting representation was a two-factor structure distinguishing peripheral criteria (salience and tolerance) from core criteria (mood modification, relapse, withdrawal, and conflict).

These findings are noteworthy because earlier BSMAS research has typically supported a unidimensional structure, whereas more recent adolescent evidence has questioned whether all six criteria carry equal clinical weight (Ciudad-Fernández et al., 2025). Although the BSMAS has previously been examined in a Swedish context, earlier evidence was based on adult samples (18 years and older) recruited largely online (Brailovskaia & Margraf, 2024). The present study therefore extends the Swedish evidence base by evaluating the scale among an adolescent, school-based sample. However, the findings should not be interpreted as showing which specific developmental, social, or contextual factors influenced psychometric performance because such factors were not directly assessed. Rather, the contribution of the present study is that the Swedish BSMAS was linguistically reviewed, culturally adapted, piloted with adolescents for clarity and age-appropriate comprehension, and then psychometrically evaluated in a school setting. This strengthens confidence that the instrument is appropriate for use in adolescent school-based research and practice, while future studies should examine whether scale performance varies according to developmental stage, social environment, school context, or patterns of social media engagement.

Importantly, the Swedish BSMAS was evaluated using both classical test theory (CTT) and an item-response framework through Rasch analysis. These complementary approaches strengthened the psychometric evaluation by examining both overall scale performance and item-level functioning. Whereas CTT supported acceptable internal consistency, factor structure, and expected associations with external variables, Rasch analysis provided additional evidence regarding item fit, response structure, differential item functioning, and measurement precision across the latent trait continuum. These findings suggest that the Swedish BSMAS functions not only as a coherent brief measure to assess PSMU, but also as an instrument with acceptable item-level performance for identifying variation in symptom severity among adolescents (Furr, 2021).

The preferred two-factor structure of the Swedish BSMAS demonstrated acceptable internal consistency, with both the peripheral and core dimensions showing satisfactory reliability. These findings suggest that the items within each dimension function coherently in the present adolescent sample. This pattern is broadly consistent with earlier studies that also supported a bidimensional BSMAS structure and reported acceptable factor-level reliability (Ciudad-Fernández et al., 2025, Fournier et al., 2023, Fournier et al., 2024). Although earlier BSMAS studies have often reported satisfactory reliability within a unidimensional framework (Bottaro et al., 2025), direct comparison with those total-score estimates should be made cautiously because the present findings did not support retaining a strictly unidimensional interpretation. Instead, the Swedish adolescent data were better represented by two closely related dimensions distinguishing peripheral from core criteria.

Regarding dimensionality, the present findings did not support retaining the Swedish BSMAS as a strictly unidimensional measure in this adolescent sample. Although much of the earlier validation literature has supported a one-factor solution (e.g., Al-Dassean and Murad, 2025, Abiddine et al., 2025, Bányai et al., 2017, Bottaro et al., 2025, Lin et al., 2017), and a recent meta-analysis summarized the broader BSMAS literature as predominantly unidimensional (Bottaro et al., 2025), the present study’s comparative CFA results indicated that a two-factor structure provided a better representation of the data. More specifically, salience and tolerance clustered as peripheral criteria, whereas mood modification, relapse, withdrawal, and conflict clustered as core criteria. This pattern is broadly consistent with recent studies showing that the six BSMAS criteria may not function as psychometrically equivalent indicators of PSMU, and that peripheral and core criteria can display distinct associations with psychological correlates (Ciudad-Fernández et al. 2025). Accordingly, the present findings suggest that, among adolescents, reliance on a single summed BSMAS score may obscure potentially meaningful distinctions between engagement-related symptoms and more clearly maladaptive involvement.

From a theoretical perspective, the present two-factor findings are not merely a statistical detail of model fit but may also inform how PSMU is conceptualized in adolescence. The components model of addiction (Griffiths, 2005), on which the BSMAS was developed, operationalizes salience, mood modification, tolerance, withdrawal, conflict, and relapse as indicators of behavioral addiction. However, earlier work suggested that tolerance and cognitive salience may reflect high engagement rather than pathology, whereas withdrawal, relapse/reinstatement, conflict, and behavioral salience appear more specific to maladaptive use (Charlton & Danforth, 2007).

Subsequent critiques have similarly emphasized that criteria-based approaches to behavioral addiction, particularly when symptoms are treated as equally weighted indicators without sufficient attention to functional impairment and distress, may risk overpathologizing intensive but otherwise functional engagement (Billieux et al., 2015, Kardefelt-Winther et al., 2017). In this context, the empirical separation of peripheral criteria (salience and tolerance) from core criteria (mood modification, relapse, withdrawal, and conflict) in the present Swedish adolescent sample is theoretically meaningful and consistent with recent work challenging the assumption that all BSMAS items function as equivalent indicators of a single latent construct (Ciudad-Fernández et al., 2025, Fournier et al., 2023, Fournier et al., 2024).

Rather than invalidating the BSMAS, these findings suggest that the scale may capture two related but distinguishable facets of social media involvement: an engagement-oriented dimension, reflected by frequent thinking about social media and increasing desire to use it, and a more maladaptive dimension, reflected by affect-regulatory use, failed reduction attempts, withdrawal-like experiences, and negative functional consequences. This distinction is important because adolescents may obtain elevated BSMAS scores for different reasons. Scores driven mainly by salience and tolerance may reflect high involvement, whereas scores driven by the co-occurrence of core criteria may more plausibly indicate distress-related and potentially impairing use.

For the BSMAS as an instrument, this suggests that its brevity and usefulness as a screening tool can be retained, and that its total score remains supported by a broad psychometric literature (Bottaro et al., 2025), while interpretation may be strengthened by inspecting whether elevated scores are driven primarily by peripheral or core criteria. The stronger associations of the core criteria with anxiety and depressive symptoms in the present study further support this interpretation. Accordingly, subscale-level or symptom-level information may complement total-score interpretation in adolescent research and early screening contexts.

Measurement invariance analyses supported the comparability of the preferred two-factor Swedish BSMAS across both gender and country of birth. More specifically, multigroup CFA provided evidence for configural, metric, and scalar invariance across boys and girls, as well as across Swedish-born and non-Swedish-born adolescents. These findings indicate that the peripheral-core structure of the scale, the strength of the item–factor relationships, and the item thresholds were sufficiently similar across groups, supporting the interpretation that the BSMAS operates in a psychometrically comparable manner among these subpopulations.

Consequently, both associations with external variables and group comparisons of latent or observed scores appear justified in the present sample. This cross-group stability strengthens confidence in the use of the Swedish BSMAS in adolescent research involving demographic comparisons. More broadly, the finding is consistent with earlier literature indicating that the BSMAS generally demonstrates satisfactory cross-group robustness, including prior evidence of gender invariance and broader cross-cultural applicability (Ruckwongpatr et al., 2024, Yue et al., 2022). Nevertheless, continued invariance testing remains important in future studies, particularly when the scale is applied in more heterogeneous adolescent samples or across other sociodemographic strata.

The SEM findings added an important layer to the interpretation of the two-factor BSMAS structure. Both peripheral and core criteria were positively associated with adolescent anxiety and depressive symptoms, but the associations were clearly stronger for the core dimension. This pattern suggests that although salience and tolerance may reflect less clinically central features than mood modification, relapse, withdrawal, and conflict, they were not psychologically neutral in this sample. At the same time, the stronger associations observed for the core criteria support their greater relevance to maladaptive or distress-related social media involvement. These findings only partly mirror recent two-factor SEM results reported by Ciudad-Fernández et al. (2025), where peripheral criteria showed inverse associations with distress indicators, whereas core criteria showed positive associations. One possible explanation is that, among the present sample, the peripheral and core factors were highly overlapping, which produced unstable coefficients in the joint model and required separate SEM estimation.

One informative finding from the network analysis was that social media use duration was directly connected to all six BSMAS items, indicating that time spent on social media is broadly related to both peripheral and core symptoms of problematic use. At the same time, social media duration showed only modest centrality in the network, suggesting that it was not among the most influential nodes in the overall symptom structure. This finding supports the view that PSMU is not reducible to time spent online alone, but also involves more qualitative features such as preoccupation, loss of control, withdrawal-like experiences, and negative functional consequences. These symptom-level findings support the view that the BSMAS items are not fully interchangeable because individual symptoms showed different patterns of association with social media use duration and internalizing symptoms. This interpretation is consistent with recent longitudinal evidence showing that addictive patterns of screen use, rather than total screen time *per se*, were associated with suicidal behaviors, suicidal ideation, and poorer mental health outcomes among early adolescents (Xiao et al., 2025).

Several limitations of the study should be acknowledged. First, the cross-sectional design precluded evaluation of test–retest reliability, temporal stability, and predictive validity of the Swedish BSMAS. Longitudinal studies are therefore needed to determine whether the scale can reliably capture change in PSMU over time and whether its scores predict later psychosocial outcomes. Second, although the Rasch analyses showed satisfactory item fit and strong item separation, person separation was more limited, particularly for the core dimension, suggesting that the scale may be less precise in distinguishing adolescents with closely similar levels of the latent trait. This is not unusual for a very brief instrument, but it should be considered when interpreting individual-level differentiation. Third, although multigroup CFA supported configural, metric, and scalar invariance across both gender and country of birth, the non-Swedish-born subgroup was relatively small, and one response category had to be collapsed for one item in the country-of-birth analysis. Replication among larger and more heterogeneous adolescent samples is therefore warranted.

Fourth, the study relied exclusively on self-report measures, which may be influenced by recall bias, social desirability, and shared method variance. Fifth, although the sample size was adequate for the applied psychometric analyses, participants were recruited from only three schools in specific regions of Sweden, which may limit generalizability to the broader Swedish adolescent population. In addition, although the sample was adolescent and school-based, the study did not directly assess developmental, social, or contextual characteristics that might shape item interpretation or psychometric performance. Therefore, future studies should examine such factors explicitly. It should also be noted that the BSMAS is a measure of general PSMU and does not capture problematic use tied to specific platforms. Future studies should therefore consider validating platform-specific instruments where relevant. For instance, recent work on the YouTube Addiction Scale (Pakpour et al 2025) highlights the growing importance of complementing general social media measures with platform-specific assessment tools.

Despite these limitations, the present study also has several important strengths. To the authors’ knowledge, the study is the first to culturally adapt and psychometrically evaluate an adolescent-specific Swedish version of the BSMAS among a school-based adolescents. In addition, the study went beyond a conventional validation approach by combining comparative CFA, measurement invariance testing, Rasch modeling, SEM, and network analysis. This multimethod design provided converging evidence not only regarding reliability and item functioning, but also regarding the scale’s dimensionality and its theoretically meaningful relationships with social media use duration, anxiety, and depressive symptoms.

## 5 Conclusion

The present study provides evidence that the Swedish version of the BSMAS is a psychometrically acceptable instrument for assessing PSMU among adolescents. Rather than supporting a strictly unidimensional interpretation, the findings favored a two-factor representation distinguishing peripheral criteria (salience and tolerance) from core criteria (mood modification, relapse, withdrawal, and conflict). Across complementary psychometric approaches, the scale showed acceptable reliability, satisfactory item functioning, and measurement invariance across gender and country of birth. In addition, the findings indicated that PSMU is not reducible to time spent on social media alone, and that core symptoms were more strongly associated with anxiety and depressive symptoms than peripheral symptoms. The Swedish BSMAS appears suitable for adolescent research, school-based studies, and early screening contexts in Sweden.

## 6 Author contribution statement

All authors listed have significantly contributed to the development and the writing of this paper.

## 7 Declaration of generative AI and ai assisted technologies in the writing process

During the preparation of this work the authors used ChatGPT 5.5 (Open AI, https://chat.openai.com/chat) to check the grammar and spelling and improve the clarity of some sentences. After using this tool, the authors reviewed and edited the content as needed and took full responsibility for the content of the published paper.

## CRediT authorship contribution statement

**Malin Jakobsson:** Writing – review & editing, Writing – original draft, Resources, Project administration, Methodology, Investigation, Data curation, Conceptualization. **Gunilla Björling:** Writing – review & editing, Data curation, Conceptualization. **Anders Broström:** Writing – review & editing, Methodology, Conceptualization. **Karina Huus:** Writing – review & editing, Methodology, Conceptualization. **Marit Eriksson:** Writing – review & editing, Methodology, Conceptualization. **Staffan Bengtsson:** Writing – review & editing, Methodology, Conceptualization. **Mark D. Griffiths:** Writing – review & editing, Conceptualization. **Amir Pakpour:** Writing – review & editing, Writing – original draft, Validation, Methodology, Formal analysis, Data curation, Conceptualization.

## Funding

There was no funding associated with this research.

## Declaration of competing interest

The authors declare that they have no known competing financial interests or personal relationships that could have appeared to influence the work reported in this paper.

## Data Availability

Data will be made available upon reasonable request from the corresponding author.

## References

[b0005] Abiddine F.Z.E., Aljaberi M.A., Alduais A., Lin C.-Y., Vally Z., Griffiths M.D. (2025). The psychometric properties of the Arabic Bergen Social Media Addiction Scale. International Journal of Mental Health and Addiction.

[b0010] Achenbach T.M., Ivanova M.Y., Rescorla L.A., Turner L.V., Althoff R.R. (2016). Internalizing/externalizing problems: Review and recommendations for clinical and research applications. Journal of the American Academy of Child & Adolescent Psychiatry.

[b0015] Ahmed O., Walsh E., Dawel A., Alateeq K., Oyarce D., Cherbuin N. (2024). Social media use, mental health and sleep: A systematic review with meta-analyses. Journal of Affective Disorders.

[b0020] Al-Dassean K.A., Murad O.S. (2025). Psychometric validation of the Jordanian version of the Bergen Social Media Addiction Scale (BSMAS). Cogent Psychology.

[b0025] American Educational Research Association, American Psychological Association, & National Council on Measurement in Education (2014). *Standards for educational and psychological testing*. American Educational Research Association.

[b0030] Andersson, J., Blomdahl, F. & Bäck, J. (2024). *Svenskarna och internet 2024*. Internetstiftelsen. Retrieved January 20, 2025, from: https://svenskarnaochinternet.se/app/uploads/2024/09/internetstiftelsen-svenskarna-och-internet-2024.pdf.

[b0035] Andreassen C.S. (2015). Online social network site addiction: A comprehensive review. Current Addiction Reports.

[b0040] Andreassen C.S., Billieux J., Griffiths M.D., Kuss D.J., Demetrovics Z., Mazzoni E., Pallesen S. (2016). The relationship between addictive use of social media and video games and symptoms of psychiatric disorders: A large-scale cross-sectional study. Psychology of Addictive Behaviors.

[b0045] Arrivillaga C., Griffiths M.D., Rey L., Extremera N. (2024). Validation of the spanish version of the bergen social media addiction scale (BSMAS) among spanish adolescents. Current Psychology.

[b0055] Astatke M., Weng C., Chen S. (2021). A literature review of the effects of social networking sites on secondary school students’ academic achievement. Interactive Learning Environments.

[b0060] Bányai F., Zsila Á., Király O., Maraz A., Elekes Z., Griffiths M.D., Andreassen C.S., Demetrovics Z. (2017). Problematic social media use: Results from a large-scale nationally representative adolescent sample. PLoS One1.

[b0065] Balcerowska J.M., Bereznowski P., Biernatowska A., Atroszko P.A., Pallesen S., Andreassen C.S. (2022). Is it meaningful to distinguish between Facebook addiction and social networking sites addiction? Psychometric analysis of Facebook addiction and social networking sites addiction scales. Current Psychology.

[b0070] Beaton D.E., Bombardier C., Guillemin F., Ferraz M.B. (2000). Guidelines for the process of cross-cultural adaptation of self-report measures. Spine.

[b0075] Beeres D., Andersson F., Vossen H., Galanti M. (2021). Social media and mental health among early adolescents in Sweden: A longitudinal study with 2-year follow-up (KUPOL study). Journal of Adolescent Health.

[b0080] Billieux J., Schimmenti A., Khazaal Y., Maurage P., Heeren A. (2015). Are we overpathologizing everyday life? a tenable blueprint for behavioral addiction research. Journal of Behavioral Addictions.

[b0085] Blomqvist I., Chaplin J.E., Henje E., Dennhag I. (2025). Psychometric properties and post-hoc CAT analysis of the Pediatric PROMIS® item banks anxiety and depressive symptoms in a combined swedish child and adolescent psychiatry and school sample. Quality of Life Research.

[b0090] Bottaro R., Griffiths M.D., Faraci P. (2025). Meta-analysis of reliability and validity of the Bergen Social Media Addiction Scale (BSMAS). Advance online publication. International Journal of Mental Health and Addiction..

[b0095] Bowen N.K., Masa R.D. (2015). Conducting measurement invariance tests with ordinal data: A guide for social work researchers. Journal of the Society for Social Work and Research.

[b0100] Bozzola E., Spina G., Agostiniani R., Barni S., Russo R., Scarpato E., Di Mauro A., Di Stefano A., Caruso C., Corsello G., Staiano A. (2022). The use of social media in children and adolescents: Scoping review on the potential risks. International Journal of Environmental Research and Public Health.

[b0105] Brailovskaia J., Margraf J. (2024). Addictive social media use during Covid-19 outbreak: Validation of the Bergen Social Media Addiction Scale (BSMAS) and investigation of protective factors in nine countries. Current Psychology.

[b0110] Casale, S., Fioravanti, G., Gori, A., Nigro, F., & Bocci Benucci, S. (2025). Investigating the role of avoidance expectancies and metacognitions about social compensation through SNSs in the pathway from psychological distress to problematic social networking sites use. *Psychological Reports*. Advance online publication. https://doi.org/10.1177/0033294125132030. 10.1177/00332941251320309 39966723

[b0115] Chaffey, D. (2025). *Global social media statistics research summary.* Smart Insight. Retrieved April 28, 2026, from: https://www.smartinsights.com/social-media-marketing/social-media-strategy/new-global-social-media-research/.

[b0120] Charlton J.P., Danforth I.D. (2007). Distinguishing addiction and high engagement in the context of online game playing. Computers in Human Behavior.

[b0125] Chen I.-H., Ahorsu D.K., Pakpour A.H., Griffiths M.D., Lin C.-Y., Chen C.-Y. (2020). Psychometric properties of three simplified chinese online-related addictive behavior instruments among mainland chinese primary school students. Frontiers in Psychiatry.

[b0130] Chen I.-H., Strong C., Lin Y.-C., Tsai M.-C., Leung H., Lin C.-Y., Pakpour A.H., Griffiths M.D. (2020). Time invariance of three ultra-brief internet-related instruments: Smartphone application-based addiction scale (SABAS), Bergen Social Media Addiction Scale (BSMAS), and the nine-item internet gaming Disorder Scale-short form (IGDS-SF9) (study Part B). Addictive Behaviors.

[b0135] Cicchetti D.V. (1994). Guidelines, criteria, and rules of thumb for evaluating normed and standardized assessment instruments in psychology. Psychological Assessment.

[b0140] Ciudad-Fernández V., Fournier L., Escrivá-Martínez T., Baños R., Zarco-Alpuente A., Billieux J. (2025). Salience and tolerance are not indicators of problematic social media use: Evidence from the Social Media Disorder Scale and the Bergen Social Media Addiction Scale. Journal of Behavioral Addictions.

[b0145] Dadiotis A., Bacopoulou F., Kokka I., Vlachakis D., Chrousos G., Darviri C., Roussos P. (2021). Validation of the Greek version of the Bergen Social Media Addiction Scale in undergraduate students. EMBnet.journal.

[b0150] Dadiotis, A., Malliari, K., Critselis, E., Bacopoulou, F., Vlachakis, D., Chrousos, G., & Darviri, C. (2024). The relationship between social media addiction, depression, stress, and anxiety: A meta-analysis. In: *International genomics, neuroscience, therapeutics and data innovation summit* (pp. 275–295). Springer Nature Switzerland. 10.1007/978-3-032-03398-7_28 41273570

[b0155] Dixon, S. (2025). *Daily time spent on social networking by internet users worldwide from 2012 to 2025*. Statista. Retrieved January 20, 2025, from: https://www.statista.com/statistics/433871/daily-social-media-usage-worldwide/.

[b0160] Fournier L., Schimmenti A., Musetti A., Boursier V., Flayelle M., Cataldo I., Starcevic V., Billieux J. (2023). Deconstructing the components model of addiction: An illustration through “addictive” use of social media. Addictive Behaviors.

[b0165] Fournier L., Schimmenti A., Musetti A., Boursier V., Flayelle M., Cataldo I., Starcevic V., Billieux J. (2024). Further evidence for the bidimensionality of the components model of addiction: A reply to Amendola (2023). Addictive Behaviors.

[b0170] Furr R.M. (2021).

[b0175] Griffiths M. (2005). A “components” model of addiction within a biopsychosocial framework. Journal of Substance Use.

[b0180] Guillemin F., Bombardier C., Beaton D. (1993). Cross-cultural adaptation of health-related quality of life measures: Literature review and proposed guidelines. Journal of Clinical Epidemiology.

[b0185] Hooper D., Coughlan J., Mullen M.R. (2008). Structural equation modelling: Guidelines for determining model fit. Electronic Journal of Business Research Methods.

[b0190] Kardefelt-Winther D., Heeren A., Schimmenti A., van Rooij A., Maurage P., Carras M., Edman J., Blaszczynski A., Khazaal Y., Billieux J. (2017). How can we conceptualize behavioural addiction without pathologizing common behaviours?. Addiction.

[b0195] Keles B., McCrae N., Grealish A. (2019). A systematic review: The influence of social media on depression, anxiety and psychological distress in adolescents. International Journal of Adolescence and Youth.

[b0200] Kross E., Verduyn P., Sheppes G., Costello C.K., Jonides J., Ybarra O. (2021). Social media and well-being: Pitfalls, progress, and next steps. Trends in Cognitive Sciences.

[b0210] Leung H., Pakpour A.H., Strong C., Lin Y., Tsai M., Griffiths M.D., Lin C., Chen I. (2020). Measurement invariance across young adults from Hong Kong and Taiwan among three internet-related addiction scales: Bergen Social Media Addiction Scale (BSMAS), Smartphone Application-based Addiction Scale (SABAS), and internet gaming Disorder Scale-short form (IGDS-SF9) (Study Part a). Addictive Behaviors.

[b0215] Li Ma B., Evans B., Kleppang A., Hagquist C. (2021). The association between screen time and reported depressive symptoms among adolescents in Sweden. Family Practice.

[b0220] Lin C.Y., Broström A., Nilsen P., Griffiths M.D., Pakpour A.H. (2017). Psychometric validation of the Persian Bergen Social Media Addiction Scale using classic test theory and Rasch models. Journal of Behavioral Addictions.

[b0225] Linacre, J. M. (2012) A user's guide to winsteps Rasch-model computer programs. Retrieved January 20, 2025, from: http://www.winsteps.com.

[b0230] Monacis L., De Palo V., Griffiths M.D., Sinatra M. (2017). Social networking addiction, attachment style, and validation of the italian version of the Bergen Social Media Addiction Scale. Journal of Behavioral Addictions.

[b0235] Montag C., Demetrovics Z., Elhai J., Grant D., Koning I., Rumpf H., Spada M., Throuvala M., Van Den Eijnden R. (2024). Problematic social media use in childhood and adolescence. Addictive Behaviors.

[b0240] Nagata J., Memon Z., Talebloo J., Li M., Low P., Shao I., Ganson K., Testa A., He J., Brindis C., Baker F. (2025). Prevalence and patterns of social media use in early adolescents. Academic Pediatrics.

[b0245] Naher L., Hiramoni F.A., Alam N., Ahmed O. (2022). Psychometric assessment of the Bangla version of the Bergen Social Media Addiction Scale. Heliyon.

[b0255] Pakpour A.H., Jafari E., Zanjanchi F., Potenza M.N., Lin C.-Y. (2025). The YouTube Addiction Scale: Psychometric evidence for a new instrument developed based on the component model of addiction. International Journal of Mental Health and Addiction.

[b0260] Parry D.A., Davidson B.I., Sewall C.J., Fisher J.T., Mieczkowski H., Quintana D.S. (2021). A systematic review and meta-analysis of discrepancies between logged and self-reported digital media use. Nature Human Behaviour.

[b0265] Patrick D.L. (2019). Many ways to skin a cat: Psychometric methods options illustrated. Journal of Patient-Reported Outcomes.

[b0270] Piccerillo L., Digennaro S. (2025). Adolescent social media use and emotional intelligence: A systematic review. Adolescent Research Review.

[b0275] Popat A., Tarrant C. (2022). Exploring adolescents’ perspectives on social media and mental health and well-being – a qualitative literature review. Clinical Child Psychology and Psychiatry.

[b0280] Rouleau R.D., Beauregard C., Beaudry V. (2023). A rise in social media use in adolescents during the COVID-19 pandemic: The French validation of the Bergen Social Media Addiction Scale in a Canadian cohort. BMC Psychology.

[b0285] Ruckwongpatr K., Paratthakonkun C., Sangtongdee U., Pramukti I., Nurmala I., Angkasith K., Thanachaisakul W., Ketchatturat J., Griffiths M.D., Kao Y.-K., Lin C.-Y. (2024). Validity, reliability, and measurement invariance of the Thai smartphone application-based addiction scale (SABAS) and Bergen Social Media Addiction Scale (BSMAS). International Journal of Mental Health Promotion.

[b0290] Sadeghi S., Shalani B., Firouzabadi S.M., Babaei Z., Arian Namazi S., Pouretemad H.R. (2025). Psychometric validation of the Farsi Bergen Social Media Addiction Scale (BSMAS) among iranian adults. BMC Public Health.

[b0295] Salari N., Zarei H., Rasoulpoor S., Ghasemi H., Hosseinian-Far A., Mohammadi M. (2025). The impact of social networking addiction on the academic achievement of university students globally: A meta-analysis. Public Health in Practice.

[b0300] Shannon H., Bush K., Villeneuve P.J., Hellemans K.G., Guimond S. (2022). Problematic social media use in adolescents and young adults: Systematic review and meta-analysis. JMIR Mental Health.

[b0305] Shin N.Y. (2022). Psychometric properties of the Bergen Social Media Addiction Scale in Korean young adults. Psychiatry Investigation.

[b0310] Sick J. (2008). Rasch measurement in language education: Part 1. JALT Testing & Evaluation SIG Newsletter.

[b0315] Stănculescu E. (2022). The Bergen Social Media Addiction Scale validity in a Romanian sample using item response theory and network analysis. International Journal of Mental Health and Addiction.

[b0320] Tesio L. (2003). Measuring behaviours and perceptions: Rasch analysis as a tool for rehabilitation research. Journal of Rehabilitation Medicine.

[b0325] Valkenburg P.M., Meier A., Beyens I. (2022). Social media use and its impact on adolescent mental health: An umbrella review of the evidence. Current Opinion in Psychology.

[b0330] Vannucci A., Simpson E., Gagnon S., Ohannessian C. (2020). Social media use and risky behaviors in adolescents: A meta-analysis. Journal of Adolescence.

[b0335] Vidal C., Lhaksampa T., Miller L., Platt R. (2020). Social media use and depression in adolescents: A scoping review. International Review of Psychiatry.

[b0345] Xiao Y., Meng Y., Brown T.T., Keyes K.M., Mann J.J. (2025). Addictive screen use trajectories and suicidal behaviors, suicidal ideation, and mental health in US youths. Journal of the American Medical Association.

[b0350] Yam C.-W., Pakpour A.H., Griffiths M.D., Yau W.-Y., Lo C.-L.-M., Ng J.M.T., Lin C.-Y., Leung H. (2019). Psychometric testing of three chinese online-related addictive behavior instruments among Hong Kong university students. Psychiatric Quarterly.

[b0355] Yue H., Zhang X., Cheng X., Liu B., Bao H. (2022). Measurement invariance of the Bergen Social Media Addiction Scale across genders. Frontiers in Psychology.

[b0360] Žmavc M., Šorgo A., Gabrovec B., Crnkovič N., Cesar K., Selak Š. (2022). The protective role of resilience in the development of social media addiction in tertiary students and psychometric properties of the Slovenian Bergen Social Media Addiction Scale (BSMAS). International Journal of Environmental Research and Public Health.

