# Supplementary material for: The Swedish version of the Bergen Social Media Addiction Scale: A psychometric evaluation among adolescents
Source: Addict Behav Rep. 2026 May 2;23:100704. doi: 10.1016/j.abrep.2026.100704 (PMC13191657; doi:10.1016/j.abrep.2026.100704)
Supplement: Supplementary Data 1 [file mmc1.docx]

**Appendix 1**

***Translation procedure***

Although Swedish-language BSMAS data have previously been reported in an adult multinational study, no adolescent-specific Swedish adaptation with cognitive testing had been conducted. The present procedure was therefore undertaken to ensure age-appropriate wording and comprehension in adolescents (Brailovskaia & Margraf, 2024).

*Step 1 – Forward translation*

Four bilingual translators, all native Swedish speakers, independently translated the original English BSMAS into Swedish. The first two translators were researchers familiar with the scale’s subject matter and experienced with psychometric instruments. The other two translators had no clinical or research backgrounds, providing fresh perspectives less influenced by academic terminology.

*Step 2 – Synthesis of translations*

The four independent Swedish translations and accompanying translator notes were collected and carefully reviewed by the research team. Minor wording differences were discussed and resolved through consensus to produce a single synthesized version. This version was then sent to two separate bilingual translators for back translation.

*Step 3 – Back translation*

Two native English speakers, blinded to the original instrument and study goals, independently back-translated the synthesized Swedish version into English. This step aimed to check the accuracy and content equivalence of the Swedish version with the original BSMAS items.

*Step 4 – Expert committee review*

An expert committee comprising healthcare professionals, researchers, translators, and adolescent representatives reviewed all materials, including the original BSMAS, forward and back translations, and translators’ notes. The committee examined semantic, idiomatic, experiential, and conceptual equivalences to produce the prefinal Swedish version of the BSMAS.

*Step 5 – Pilot testing of the prefinal version*

The prefinal Swedish BSMAS was pilot tested with 35 adolescents (19 females) aged 13 to 16 years. Each participant completed the scale and then participated in cognitive interviews to explore their understanding of each item and response option. Adolescents identified several words that were too formal or difficult, providing constructive suggestions to improve clarity and age-appropriateness while maintaining the intended meaning. In response, the wordings in Items 3 and 5 were changed slightly by the expert committee, resulting in the finalized version used in the present study. The expert committee revised the scale, accordingly, resulting in the finalized version used in the present study.

**Appendix 2: Final version of the Swedish BSMAS**

**Sociala medier** (så som *Instagram, Tiktok, Snapchat, X, Facebook* eller liknande)

| **Hur ofta under det senaste året har du…** | | | | | |
| --- | --- | --- | --- | --- | --- |
|  | **Väldigt sällan**  **1** | **Sällan**  **2** | **Ibland**  **3** | **Ofta**  **4** | **Väldigt ofta**  **5** |
| …har du tänkt på eller längtat efter att använda sociala medier när du inte haft möjlighet att vara inne på dem | □ | □ | □ | □ | □ |
| …känt att du vill använda sociala medier mer och mer | □ | □ | □ | □ | □ |
| …använt sociala medier för att inte tänka på jobbiga saker (ex. olika personliga problem) | □ | □ | □ | □ | □ |
| …försökt att använda sociala medier mindre utan att det har fungerat | □ | □ | □ | □ | □ |
| …blivit orolig eller mått dåligt när du inte haft möjlighet att använda sociala medier | □ | □ | □ | □ | □ |
| …använt sociala medier så mycket att det påverkat dina studier/skolarbete negativt | □ | □ | □ | □ | □ |
